# Comment on “Electrophysiological and Biochemical Evaluations of Neuropathy Risk in Oral Levodopa versus Levodopa/Carbidopa Intestinal Gel Treatment”

**DOI:** 10.1055/s-0046-1825519

**Published:** 2026-09-15

**Authors:** Kishankumar Mahida, Snehal Rajendra Jagtap

**Affiliations:** 1 Dr. D. Y. Patil Medical College Hospital and Research Centre, Dr. D. Y. Patil Vidyapeeth (Deemed-to-be-University), Pimpri, Pune, 411018, Maharashtra, India.; 2 Dr. D. Y. Patil Medical College Hospital and Research Centre, Dr. D. Y. Patil Vidyapeeth (Deemed-to-be-University), Pimpri, Pune, 411018, Maharashtra, India.

Dear Editor,

We read with great interest the prospective analysis by Erdem et al. 1 comparing electrophysiological and biochemical markers of neuropathy risk in Parkinson's disease patients treated with oral levodopa or levodopa/carbidopa intestinal gel. The integration of nerve-conduction studies with homocysteine and vitamin profiling provides a clinically-oriented multidimensional framework, and the standardized neurophysiology protocol strengthens internal consistency. However, several interpretive aspects merit closer examination.

The attribution of greater neuropathy risk to levodopa/carbidopa intestinal gel may partially reflect exposure intensity rather than delivery route. Patients receiving intestinal gel had markedly higher levodopa equivalent daily dose, exceeding the oral group by more than 1,100 mg per day. Such dose asymmetry introduces a pharmacometabolic gradient in which homocysteine elevation and axonal stress could be dose-mediated rather than route-specific. 2

Clinically, this distinction matters because treatment selection may shift toward or away from intestinal infusion based on perceived modality risk rather than cumulative levodopa burden. Dose-normalized modeling or stratification by levodopa equivalent daily dose bands would help clarify whether the observed electrophysiological differences persist after exposure harmonization.

Interpretation of the neurophysiological signal also warrants refinement. The analysis relies primarily on unilateral nerve conduction studies and median comparisons across multiple nerves; yet, the pattern described is heterogeneous, with some parameters differing while others remain similar among groups. In Parkinson's disease, subclinical polyneuropathy typically follows a length-dependent and symmetric pattern. 3 When abnormalities cluster in selected nerves without a clear distal gradient, the possibility of physiological variability or focal vulnerability should be considered. From a clinical-decision standpoint, establishing a composite neuropathy severity index or applying predefined electrophysiological diagnostic criteria could improve confidence that the observed changes represent diffuse polyneuropathy rather than parameter-level fluctuation.

The biochemical correlations, although biologically-plausible, are presented in a largely cross-sectional framework that limits causal interpretation. Moderate associations regarding homocysteine and conduction metrics do not establish temporal directionality between metabolic disturbance and axonal dysfunction. 4 Clinicians may otherwise infer that biochemical correction alone will modify neuropathy trajectory. Incorporating longitudinal follow-up with repeated homocysteine measurements and nerve-conduction studies, or testing mediation models linking levodopa exposure to metabolic change and electrophysiological decline, would strengthen translational inference.

We commend the authors for advancing an integrated electrophysiological and biochemical perspective on neuropathy risk in advanced Parkinson's disease. Future longitudinal, dose-matched investigations incorporating composite neuropathy definitions and temporal metabolic profiling may further refine risk stratification and guide individualized levodopa delivery strategies.
